# Supplementary material for: Prevention and Killing Efficacy of Carbapenem Resistant Enterobacteriaceae (CRE) and Vancomycin Resistant Enterococci (VRE) Biofilms by Antibiotic-Loaded Calcium Sulfate Beads
Source: Materials (Basel). 2020 Jul 22;13(15):3258. doi: 10.3390/ma13153258 (PMC7436038; doi:10.3390/ma13153258)
Supplement: Supplementary file 1 [file materials-13-03258-s001.pdf]

materials

Supplementary Materials: Prevention and Killing Efficacy of Carbapenem Resistant *Enterobacteriaceae* (CRE) and Vancomycin Resistant *Enterococci* (VRE) Biofilms by Antibiotic-Loaded Calcium Sulfate Beads

Paul Stoodley [1,2,3], Jacob Brooks [1], Casey W. Peters [1], Nan Jiang [1], Craig P. Delury [4], Phillip A. Laycock [4], Sean S. Aiken [4] and Devendra H. Dusane [1,5,*]

[1] Department of Microbial Infection and Immunity, The Ohio State University, Columbus, OH 43210, USA; Paul.Stoodley@osumc.edu (P.S.); jacob.brooks@osumc.edu (J.B.); caseywilliampeters@gmail.com (C.W.P.), nian.jaing@osumc.edu (N.J.)
[2] National Centre for Advanced Tribology, Faculty of Engineering and Institute for Life Sciences, University of Southampton, SO17 1BJ Southampton, UK
[3] Department of Orthopaedic, The Ohio State University, Columbus, OH 43210, USA
[4] Biocomposites Ltd., Keele Science Park, Keele, ST5 5NL Staffordshire, UK; cpd@biocomposites.com (C.P.D.); pl@biocomposites.com (P.A.L.); sa@biocomposites.com (S.S.A.)
[5] Center for Clinical and Translational Research, The Research Institute at Nationwide Children's Hospital, 700 Children's Drive, Columbus, OH 43205, USA
* Correspondence: devendra.dusane@nationwidechildrens.org

Table 1. Multivariate analysis (ANOVA) of prevention and killing treatment groups.

**Parameters**	**Prevention Group**		**Killing Group**	
	**Test Statistics (F)**	***p* Value**	**Test Statistics (F)**	***p* Value**
Corrected model	40.658	**< 0.001**	30.874	**< 0.001**
Exposure time	139.609	**< 0.001**	63.883	**< 0.001**
Bacteria strain	66.330	**< 0.001**	13.234	**< 0.001**
Treatment	555.882	**< 0.001**	305.467	**< 0.001**
Exposure time × Bacteria strain	8.743	**< 0.001**	24.298	**< 0.001**
Exposure time × Treatment	6.690	**< 0.001**	19.147	**< 0.001**
Bacteria strain × Treatment	11.380	**< 0.001**	6.665	**< 0.001**
Exposure time × Bacteria strain × Treatment	2.088	**0.001**	2.220	**0.003**

Table 2. Biomass and thickness of biofilms in prevention group analyzed by COMSTAT.

**Prevention Biomass ($\mu m^3/\mu m^2$)**		**KP 1705**	**KP 2146**	**KP 2524**	**EF 51299**	**Prevention Thickness (μm)**		**KP 1705**	**KP 2146**	**KP 2524**	**EF 51299**
Day 1	**Control**	2.59	4.59	1.93	3.88	Day 1	**Control**	8.90	9.52	9.73	10.40
	**GV**	2.05	2.11	1.50	1.44		**GV**	3.72	4.14	4.35	3.72
	**RM**	0.98	0.15	0.11	1.11		**RM**	2.69	2.48	3.52	2.90
	**RV**	1.58	0.32	0.50	1.07		**RV**	3.10	3.93	4.04	5.80
	**VT**	2.50	1.04	1.25	0.86		**VT**	5.38	8.28	3.10	3.93
Day 3	**Control**	2.35	2.77	2.09	2.42	Day 3	**Control**	4.55	4.55	4.55	1.00
	**GV**	5.45	0.39	0.42	0.46		**GV**	12.22	3.72	1.00	1.86
	**RM**	0.25	0.85	0.26	0.13		**RM**	1.00	1.00	1.00	3.93
	**RV**	1.23	2.32	0.27	0.88		**RV**	2.69	6.00	1.45	4.35
	**VT**	1.48	0.37	0.30	1.75		**VT**	3.72	1.00	1.24	3.25

Table 3. Biomass and thickness of biofilms in killing group analyzed by COMSTAT.

Killing Biomass ($\mu m^3/\mu m^2$)		KP 1705	KP 2146	KP 2524	EF 51299	Killing Thickness (μm)		KP 1705	KP 2146	KP 2524	EF 51299
Day 1	**Control**	3.51	3.64	3.68	5.65	Day 1	**Control**	6.01	3.94	9.74	6.01
	**GV**	2.32	2.05	2.07	2.59		**GV**	8.08	3.94	6.01	3.25
	**RM**	2.71	2.26	0.65	0.85		**RM**	3.94	4.76	2.21	1.04
	**RV**	1.80	2.44	0.83	0.58		**RV**	5.59	2.94	2.35	1.00
	**VT**	2.07	2.42	1.85	2.26		**VT**	4.97	6.01	5.59	1.00
Day 3	**Control**	4.38	4.07	2.11	8.10	Day 3	**Control**	1.4297	1.82	2.48	3.82
	**GV**	3.53	3.37	1.51	1.35		**GV**	0.4438	1.98	0.44	0.31
	**RM**	1.05	0.22	0.10	0.27		**RM**	0.3289	0.48	0.44	0.28
	**RV**	0.73	0.15	0.43	0.14		**RV**	0.2211	0.19	0.24	0.24
	**VT**	0.91	2.68	2.45	0.88		**VT**	0.3550	0.52	0.21	0.32

© 2020 by the authors. Submitted for possible open access publication under the terms and conditions of the Creative Commons Attribution (CC BY) license (http://creativecommons.org/licenses/by/4.0/).
